# The emerging role of extracellular vesicles in viral transmission and immune evasion

**DOI:** 10.3389/fimmu.2025.1634758

**Published:** 2025-09-01

**Authors:** Muhammad Saleem, Chieh-Wei Chang, Abdul Qadeer, Mohammed Asiri, Fuad M. Alzahrani, Khalid J. Alzahrani, Khalaf F. Alsharif, Chien-Chin Chen, Shahid Hussain

**Affiliations:** ^1^ Department of Biotechnology, Kohsar University, Murree, Pakistan; ^2^ Division of General Surgery, Department of Surgery, Ditmanson Medical Foundation Chia-Yi Christian Hospital, Chiayi, Taiwan; ^3^ Department of Cell Biology, School of Life Sciences, Central South University, Changsha, China; ^4^ Department of Clinical Laboratory Sciences, College of Applied Medical Sciences, King Khalid University, Abha, Saudi Arabia; ^5^ Department of Clinical Laboratories Sciences, College of Applied Medical Sciences, Taif University, Taif, Saudi Arabia; ^6^ Department of Pathology, Ditmanson Medical Foundation Chia-Yi Christian Hospital, Chiayi, Taiwan; ^7^ Department of Cosmetic Science, Chia Nan University of Pharmacy and Science, Tainan, Taiwan; ^8^ Doctoral Program in Translational Medicine, National Chung Hsing University, Taichung, Taiwan; ^9^ Department of Biotechnology and Bioindustry Sciences, College of Bioscience and Biotechnology, National Cheng Kung University, Tainan, Taiwan

**Keywords:** extracellular vesicles, viral infections, immune modulation, therapeutic, biomarkers, nanocarriers

## Abstract

Extracellular vesicles (EVs) are membrane-bound structures that serve as major mediators of intercellular communication, playing a crucial role in various physiological and pathological processes. These membrane-bound vesicles are involved in several biological processes and are essential because they play a vital role in regulating viral infections. Given the global burden of viral diseases, understanding the interaction between EVs and viruses is crucial for the development of novel diagnostic tools and therapeutic strategies. This review provides a comprehensive examination of the structure and nature of EVs, as well as their biogenesis and molecular components, distinguishing between exosomes, microvesicles, and apoptotic bodies. We discuss the relationship between EVs and viral diseases, as well as their roles in viral pathogenesis and the dissemination of infections. Moreover, based on the ability of viruses to modulate host immune responses at both the innate and adaptive levels, the involvement of EVs in immune evasion is described. Additionally, the ability of EVs to diagnose viral illnesses and their therapeutic applications, such as using EVs for vaccines, immunotherapy, and the delivery of antiviral drugs, will also be discussed. Various viral diseases, including HIV, hepatitis B and C, and influenza, as well as emerging viruses such as SARS-CoV-2, are reviewed to capture the multifaceted functions of EVs in viral diseases. Finally, the review discusses the limitations of EV research, factors that affect the standardization of the technique, and the outlook for clinical applications. Based on a synthesis of current literature knowledge, this review aimed to identify and highlight the potential of EVs as diagnostic and therapeutic agents in the prevention and treatment of viral infections, thereby paving the way for further research and innovation.

## 1 Introduction

**Figure 1:**
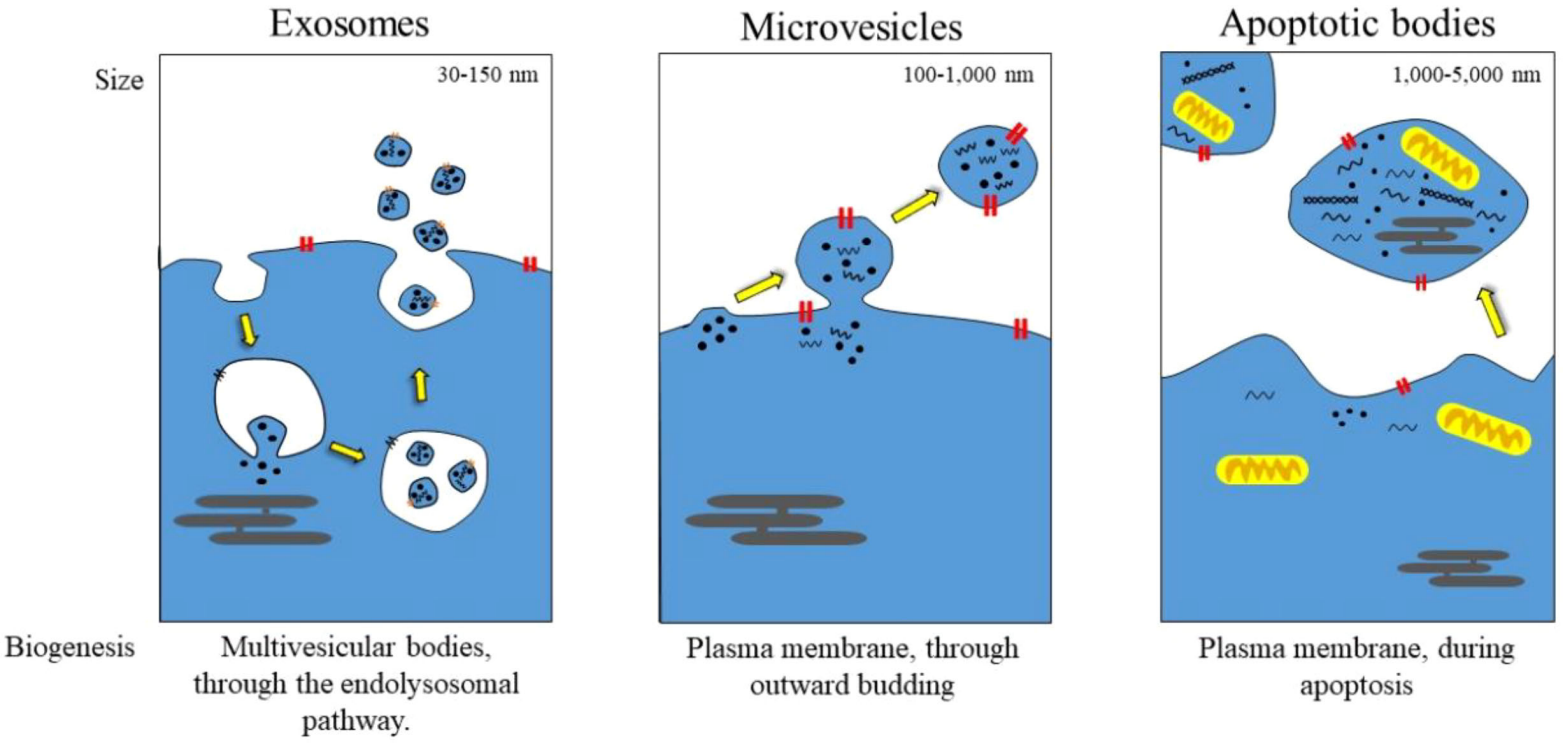
Schematic representation of EVs release.

Extracellular vesicles (EVs) are membrane-bound structures found in various cell types and are found in body fluids, including the blood, saliva, and cerebrospinal fluid (1). These vesicles also play a crucial role in the exchange of materials between cells, including proteins, lipids, and nucleic acids. EVs are primarily categorized into exosomes, microvesicles, and apoptotic bodies based on their size, origin, and biogenesis (as shown in **Figure 1**). Lipids play a crucial role in various physiological processes, including immunity, tissue regeneration, and cellular homeostasis (2–4).

Infections with viruses continue to be a significant threat to global health because they cause a substantial burden of disease and death globally. Examples of viral infections such as HIV, hepatitis B, and C, influenza, and new viruses like SARS-CoV-2 have severe social and economic impacts. Many of these infections involve complex interactions between the virus and host cells, which are fundamental to immunology, disease development, and treatment responses. Although antiviral drugs and vaccines have been developed, issues like drug resistance, high mutation rates, and limited access to healthcare remain challenges (5–8). Recently, EVs have gained attention regarding their role in viral infections. Viral vectors also use EVs to transport viruses, evade the host immune response, and boost viral replication within the host. They can carry viral proteins, RNA, and DNA, mimicking infectious particles and influencing immune signaling (9, 10). Furthermore, during viral infections, the EVs released reflect the physiological status of the infected cells, making them potential biomarkers for diagnosing, monitoring, and prognosis of diseases (9). Because they can easily cross biological barriers, EVs are also being considered for therapeutic purposes as drug carriers and in vaccine development (11, 12).

This review offers a comprehensive overview of the complex roles of EVs in viral infections. Its main objectives are: 1. To explore the synthesis, characterization, and biogenesis of EVs. 2. To examine their role in viral pathogenesis, especially in viral transmission and modulation of the host immune response. 3. To emphasize the diagnostic and therapeutic potential of EVs, including their use in biomarker discovery, antiviral drug delivery systems, and vaccine development. 4. To analyze the role of EVs in specific viral infections such as HIV, hepatitis virus, influenza virus, and emerging pathogens like SARS-CoV-2. 5. To address the current challenges in EV research, with a focus on standardizing isolation techniques and transitioning EV-based technologies into clinical practice. By bridging basic research with clinical applications, this review highlights the transformative potential of EVs in enhancing the understanding, diagnosis, and treatment of viral infections. It outlines future directions for developing EV-based therapies.

## 2 Types and biogenesis of EVs

### 2.1 Classification of EVs

EVs are a large family of membrane-enclosed extracellular nanoparticles secreted by cells into their surrounding environment. They are typically classified based on physical features such as size and density, biochemical composition, or cell of origin; however, the distinctness of subtypes can be somewhat ambiguous due to overlapping characteristics (13, 14). The International Society for Extracellular Vesicles (MISEV2018) has launched guidelines to facilitate a more uniform classification of EVs, requiring thorough multi-parametric characterization to prevent mislabeling (15). The MISEV 2023, given new nomenclature recommendations, separation protocols, EV release/uptake assays, and *in vivo* protocols. These guidelines strongly emphasize strict reporting of EV purity (including non-vesicular contaminants), detection instrument limits, dose–response and time-course functional assays, and the use of appropriate non-EV biological controls (16). Despite these efforts, many reviewed studies still fall short of full compliance, highlighting the need for transparent reporting. EVs, including exosomes, microvesicles, and apoptotic bodies, are recognized into three prominent subtypes, each with different functions in health and disease (17).

#### 2.1.1 Exosomes

Exosomes typically range in size from 30–150 nm and are among the smallest types of EVs. While traditionally believed to originate from endosomes, recent research has clarified rather than definitively confirmed their precise origin (18, 19). They are also enriched in tetraspanins [e.g., CD9, CD63, and CD81], generally used for canonical identification, although detecting these is not an exclusive feature, as tetraspanins appear on other EV subtypes (17, 20, 21). Moreover, exosomes can also package heat shock proteins (HSP70, HSP90) and endosomal sorting complexes (ESCRT-related proteins such as Alix and TSG101). Their membrane composition reflects their endosome maturation degree, with a higher cholesterol and sphingomyelin content than in other EVs (22, 23). Exosomes play a central role in intercellular communication by enabling bioactive molecules (proteins, lipids, nucleic acids such as mRNA, miRNA, and non-coding RNA) to be transmitted from one cell to another (24, 25). Virtually all aspects of immune regulation, cancer progression, and neurodegenerative diseases are influenced by them. Various cell types produce exosomes that can be released into the surrounding environment, including cancer cells. For instance, tumor-derived exosomes can prime primary organs and distant tissues through the dissemination of oncogenic miRNAs (26). Conversely, neuronal exosomes are released in Parkinson’s disease to facilitate peripheral protein transport via α-synuclein (27).

#### 2.1.2 Microvesicles (MVs, ectosomes)

Microvesicles (100–1000 nm) are formed by the direct outward budding of the plasma membrane, setting them apart from exosomes (28). Unlike exosomes, which are relatively uniform in size, MVs exhibit a greater level of heterogeneity, partly due to differences in their release kinetics (29). Often, their surface markers reflect the composition of the parent cell membrane, such as integrins, selectins, and phosphatidylserine, which is exposed at the distal end of vesicles during their formation (30). The roles of MVs in thrombosis, inflammation, and cancer are increasingly recognized. For example, platelet-derived MVs promote coagulation by shedding pro-coagulant phospholipids, while cancer cell-derived MVs facilitate angiogenesis and induce metastasis through transforming metalloproteinases and oncogenic receptors (31, 32). Their remarkable functional plasticity is due to their ability to sequester and deliver a diverse range of molecular cargo, including signaling proteins and genetic material, back to the recipient cells.

#### 2.1.3 Apoptotic bodies

The largest EVs (1–5 µm) are released exclusively from dying cells undergoing apoptosis (33, 34). They are characterized by a morphological hallmark of membrane blebbing and severe breakdown in the cytoskeleton, forming vesicles that contain nuclear remnants, organelles, and fragmented cellular components (35). Key markers include annexin V, which detects if phosphatidylserine faces outward, and caspases, which indicate apoptosis (36). Until recently, they were thought to be nothing more than debris for phagocytic clearance; however, there is now increasing evidence that apoptotic bodies play a role in immune modulation and tissue homeostasis (37). In another context, they support the uptake of autoantigens, which could lead to autoimmunity. Conversely, in a different context, they aid tissue repair by delivering growth factors and mRNA to neighboring cells (38).

#### 2.1.4 Emerging and non-canonical EV subtypes

In addition to these traditional categories, recent advances in EVs have identified unique vesicular populations that were not previously classified. For example, migrasomes, large vesicles (up to 3 µm) observed in migrating cells, contain chemokines and growth factors, indicating a potential role in spatial signaling during development or tissue repair (39, 40). Released by aggressive metastatic cancer cells, Oncosomes are up to 10 µm in size and loaded with oncogenic cargo that promotes invasion and drug resistance. Exomeres (greater than 50 nm) are another population on the smaller end of the size spectrum. However, their classification as EVs is still debated due to their entirely different metabolic properties as a nanoparticle species (41).

### 2.2 Biogenesis of different EV types

EVs are produced through distinct but sometimes overlapping cellular pathways, which can contribute to the structural and functional diversity. Their formation involves specialized molecular machinery that governs their size, cargo composition, and eventual secretion into the extracellular environment. Among the different types of EVs, exosomes are the most well-characterized in terms of EV subpopulation, characterized by their consistent size and morphology. They are derived from the endosomal system, beginning with the inward budding of the endosomal membrane, which leads to the formation of intraluminal vesicles (ILVs) within multivesicular bodies (MVBs) (42). This budding process is primarily driven by the endosomal sorting complex required for transport (ESCRT), a group of conserved protein complexes that selectively sort and package cargoes, such as ubiquitinated proteins and nucleic acids, into newly developed ILV. Besides ESCRT-dependent mechanisms, other factors, such as ceramide, lipids, and tetraspanin-enriched microdomains, also contribute to ILV formation, highlighting the flexibility in exosome biogenesis. Once MVBs are fully formed, they can either fuse with lysosomes for content degradation or be moved toward the plasma membrane with the help of Rab GTPases (e.g., Rab27a/b) and SNARE proteins, ultimately releasing ILVs into the extracellular space as exosomes (43–45).

In sharp contrast, microvesicles (MVs), which are produced by outward budding of the plasma membrane, are driven by cytoskeletal rearrangements and mechanical signals with Ca^2+^ dependent downstream communication (46). High intracellular calcium levels then activate enzymes (e.g., flippases and scramblases) that change the distribution of phosphatidylserine from the inside to the outer leaflet, a characteristic feature of MVs surfaces (46). The simultaneous contraction of actin-myosin filaments, operating via RhoA/ROCK pathways, generates the force needed for membrane protrusion and fission. Unlike exosomes, MVs contain cytoplasmic components nonspecifically like the mother cell into the offspring; this often reflects the cell’s actual state, including metabolites, cytosolic proteins, and even organelles (47). The size and component heterogeneity reflect the diverse construction of these vesicles, which can be increased under stress or disease conditions, such as cancer or inflammation.

Apoptotic bodies are the most considerable EV fraction, formed exclusively during cell apoptosis. During apoptosis, caspase activation leads to the widespread denaturation and breakdown of the cell’s cytoskeleton, ultimately resulting in the cell’s demise through blebbing and fragmentation. Apoptotic bodies contain blebs with nuclear remnants, organelles, and degraded genomic DNA, distinguishing them from all other EVs (34, 35). Their release is a final event, often acting as an “eat me” signal for phagocytes. However, recent findings suggest that apoptotic bodies may also carry signaling function as they sometimes contain intact, functional molecules such as growth factors, regulatory RNAs, that can influence neighboring cells (48, 49).

Emerging EV subtypes have unique biogenesis mechanisms that go beyond these classical pathways. For example, migrasomes are generated when vesicles and cytoskeleton elements are pinched off at retraction fibers in migrating cells as they move forward. Oncosomes released by metastatic cancer cells originate from abnormal membrane shedding, a feature of oncogenic metabolic reprogramming, often containing cytoplasmic material that enables their extensive dissemination (50, 51). Smaller nanoparticles (exomeres; without a lipid bilayer) also challenge traditional EV concepts, as exomeres arise from non-vesicular secretory routes, although their biogenesis is not well understood (52).

Although this crosstalk indicates other complexities of EV biology, although exosomes and MVs are constantly released, this might result from transcriptional stress, hypoxia, or disease influencing cargo selection and secretion rates (53). Apoptotic bodies are closely linked to cell death. Although their utility overlaps with that of EVs, this complicates their classification. Live-cell imaging and CRISPR-based screens are now beginning to dissect these pathways, identifying conserved molecular regulators that connect distinct biogenesis mechanisms, such as TSG101 or ARF6 (53, 54). Nonetheless, the question of cargo sorting remains open. Additionally, membrane lipids are vital, and it is unclear how EV subtypes represent distinct populations versus a smear of vesiculation events.

## 3 Integration of EV omics data and public databases

High-throughput omics technologies such as transcriptomics, proteomics, lipidomics, and metabolomics have enabled the detailed annotation of EV cargo. Hundreds of thousands of entries for proteins, RNAs, lipids, and metabolites from a wide variety of EV types now exist in public databases, including ExoCarta, Vesiclepedia, and EVpedia (55). ExoCarta, primarily focused on exosomal studies, contains manually curated datasets of proteins, lipids, and RNA from various cell types and body fluids (56). Vesiclepedia broadens this scope to include all EV subtypes, such as exosomes, microvesicles, apoptotic bodies, and even recently identified extracellular particles like exomeres and supermeres, all featuring metadata like isolation protocols, EV METRIC scores, and sample origins (57). EVpedia enhances these databases by adding orthologue prediction, gene ontology enrichment, and network analysis tools for systematic omics interpretation (58). These tools are essential for placing EV cargo findings in the context of viral infection research. They enable researchers to compare fold change estimates or abundance measures (such as normalized read counts or spectral intensities) directly between infected and control groups, thereby helping to address reviewers’ concerns about biological significance. For example, miRNA associated with SARS-Co-V-2 infection can be assessed for detection frequency across datasets, relative EV abundance in plasma, and putative targets or pathways through GO enrichment analysis. Tools like FunRich, EV TRACK, and EV-TRACK linked EV METRIC provide standardized data querying and analysis capabilities across studies (59).

When viral cargo (e.g., miR-148a or Nef protein) is detected in patient-derived EVs, Vesiclepedia provides relative fold-change information across multiple datasets, helping determine whether measured levels are unusual or within expected ranges. Researchers can download abundance estimates, view experimental metadata (including EV isolation methods: UC, SEC, density gradient), and examine EV METRIC scores to assess technical strength (60). Many recent studies include these platforms to validate presumptive EV biomarkers, reducing reliance on mere descriptive trends. There are significant limitations: omics data sets vary in isolation protocols, quantification platforms, and reporting standards, often including heterogeneous metadata that complicates cross-study meta-analysis. The correlation between transcriptomic and proteomic levels is modest (r ~ 0.4), and lipidomic/metabolomic coverage is less comprehensive (60). EV-TRACK community annotations highlight that average EV metric scores remain low (~41%), indicating variable methodological transparency (60).

Theoretical models that combine multi-omic EV data sets enable predictive modeling of EV function in viral pathogenesis. For example, integrating miRNA and mRNA cargo distributions with network analysis helps predict host pathways influenced by EVs on viral infection. Ortholog matching across species can be used to validate conservation of EV markers or regulatory RNAs. Ultimately, combining these database-derived insights adds mechanistic depth, enhances quantitative understanding, and increases translational relevance in the context of EV viral infections.

## 4 Molecular composition of EVs

EVs are a diverse group of molecular cargo, which is specific to the source cell type and biological function. The membrane-bound nanoparticles deliver proteins, lipids, nucleic acids, and metabolites between cells (Data in **Table 1** indicate the composition or delivery of different particles), facilitating intercellular communication that influences both normal and disease-related processes. The molecular content of EV subtypes, such as exosomes, microvesicles, and apoptotic bodies, as well as EVs derived from various cell types or states, varies significantly (66, 67). This complexity in the molecular makeup of EVs provides essential insight into EV biogenesis, functions, and potential uses as therapeutic agents.

**Table 1:** Classification and biogenesis of extracellular vesicles.

Molecular class	Exosomes (30–150 nm, endosomal origin)	Microvesicles(100–1000 nm, plasma membrane origin)	Apoptotic Bodies(1000–5000 nm, apoptotic cell disintegration)	Reference
Lipids	- Cholesterol, sphingomyelin, ceramide - Ceramide critical for MVB formation	- Phosphatidylserine (PS) externalization - Glycosphingolipids, gangliosides	- Oxidized lipids - High PS exposure as “eat-me” signal	(61, 62)
Proteins	- Tetraspanins: CD9, CD63, CD81 - ESCRT proteins: TSG101, Alix - HSP70, HSP90 - Rab GTPases, annexins	- Integrins, selectins, flotillins - EGFR (e.g., in cancer MVs) - Cytoskeletal protein	- Histones, nuclear proteins - Activated caspases - Organelle fragments	(15, 63)
Cell-Specific Proteins	- Synaptophysin (neuronal) - MHC I/II (immune) - Mutant KRAS, MET, MMP2/9 (tumor-derived)	- Cell surface receptors - Signaling molecules (context-dependent)	- Reflect parent cell content - Apoptotic-specific proteins	(17, 64)
Nucleic Acids	- miRNAs: miR-21, miR-155 - mRNA, lncRNA, circRNA - mtDNA	- mRNA, lncRNA - Chromosomal DNA fragments	- Chromatin and mitochondrial DNA - Potential autoantigens	(65)
Metabolites & Small Molecules	- ATP, amino acids, lactate - Prostaglandins, cytokines	- Tumor metabolites - Immunomodulatory molecules	- Residual cell metabolites - Death-associated signals	(59)

### 4.1 Protein cargo of EVs

EVs are proteins involved in vesicle formation, trafficking, and uptake within vesicles. Exosomes are identified by tetraspanins (CD9, CD63, CD81) that are organized into functional microdomains on the EVs surface and are essential for cargo selection, recognition, and recipient cell interactions. Common exosomal proteins include ESCRT components (Alix and TSG101), Heat shock proteins (Hsp70 and Hsp90), and fusion membrane regulators (Rab GTPases and annexin (22, 73). These proteins determined both the identity of exosomes and their release/uptake. While microvesicles typically contain a diverse array of plasma membrane proteins, such as integrins, selectins, and flotillins, similar to those directly budding from the cell surface. They can also carry cell-specific receptors, such as the epidermal growth factor receptor (EGFR), in exosomes derived from cancer cells. These may include MITF expression and are believed to induce signaling pathways in recipient cells (74). It is hard to mistake apoptotic bodies for other EVs due to their content of cell debris, including histones and nuclear proteins, as well as the confirmed release of activated caspase from fragmented cells. Besides these common markers, EVs contain cell-type-specific proteins that reflect the characteristics of their parental cells. For example, neuronal EVs are enriched with synaptophysin, a synaptic protein, and EVs from the immune system display major histocompatibility complex (MHC) molecules involved in antigen processing (75, 76). Tumor-derived EVs are especially rich in oncoproteins (eg, mutant KRAS, MET) and proteases (MMP2/9), which contribute to invasive and metastatic properties. Notably, the proteomic landscape of EVs offers excellent potential as yet-to-be-undetermined biomarkers of diseases, including cancer, neurodegenerative disorders, and cardiovascular injuries (**Table 1**).

### 4.2 Lipid composition of EVs

The lipid bilayers of EVs are not just static structural components, but also serve as the programming machinery that regulates vesicle robustness, bioactivity, targeting, and functionality. The exosomal membrane is extremely rich in cholesterol, sphingomyelin, and ceramide, which are relatively complex and less degradable (77). Ceramide plays a key role in exosome biogenesis by facilitating the budding of intraluminal vesicles into multivesicular bodies and interacting with other proteins that promote exocytosis. The lipid profile of microvesicles is less consistent than that of EVs, because it mainly reflects the apolar phospholipid asymmetry across the parental plasma membrane (78). Notably, platelet-derived microvesicles contain a high amount of phosphatidylserine due to its externalization, which acts as a phagosomal recognition marker and as a procoagulant biomarker. They also have elevated levels of glycosphingolipids and gangliosides to influence EV interaction with target cells (79). Variations in the lipid content of EVs impact their native functions and have implications for therapeutic interventions, such as lipid modification on EVs for drug delivery (**Table 1**) (80).

### 4.3 Nucleic acids in EVs

Although EVs are involved in horizontal gene transfer, they contain a wide variety of nucleic acids, including messenger RNA (mRNA), microRNA (miRNA), long non-coding RNA (lncRNA), and even mitochondrial DNA (mtDNA). Exosomal miRNAs, which are strongly linked to various aspects of gene expression in recipient cells, such as miR-21 and miR-155, play a roles in cancer, immune modulation, and tissue repair (81). These miRNAs are typically protected from degradation via ribonucleoprotein complexes, ensuring their functional delivery. Microvesicles and Annexin V apoptotic bodies can carry larger amounts of the genetic material; they are capable of transporting chromosomal DNA fragments that may be involved in oncogenic transformation or autoimmune responses (35, 82). For example, tumor EVs contain oncogenic DNA sequences (e.g., amplified EGFRvIII) that can integrate into the genome of the recipient cell, resulting in malignant transformation. Increasing evidence shows that EVs can carry nucleic acids, such as reporter genes, which may be helpful in liquid biopsies for early cancer detection (**e.g., Data in** **Table 1**).

### 4.4 Metabolites and other small molecules

Recent research suggests that EVs may also play a role in systemic metabolic functioning by releasing metabolites, such as glucose, lactic acid, and amino acids, which can affect the metabolic state of recipient cells. In the stromal cells, tumor-derived EVs often carry signaling molecules associated with the Warburg effect, such as elevated levels of metabolites like lactate that promote glycolysis and support tumor growth. EVs also contain signaling molecules such as prostaglandins and cytokines, which can modulate inflammation (**Table 1**) (83, 84).

## 5 EVs in viral pathogenesis and transmission

**Figure 2:**
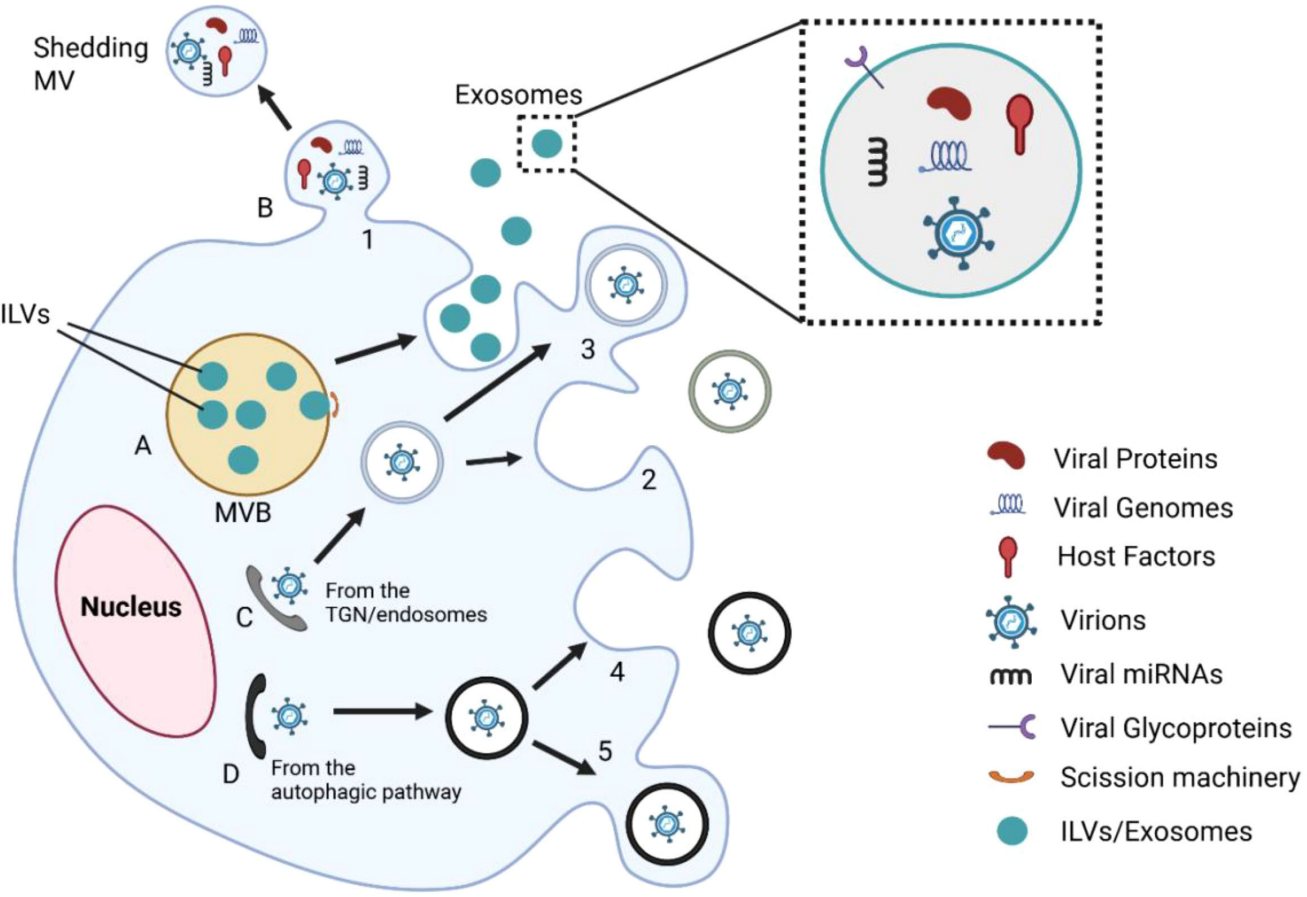
Integrative model of EV-mediated interactions during viral infection. This figure illustrates several EV-mediated processes that lead to viral infection. **(A)** Viral elements, such as proteins, genomes, and miRNAs, are packaged into intraluminal vesicles (ILVs) inside multivesicular bodies (MVBs). **(B)** Microvesicles that are released directly from the plasma membrane also contain viral material. **(C, D)** Viral particles are transferred from the trans-Golgi network or autophagic routes and packaged into vesicles (1–5). EVs facilitate viral dissemination, immune modulation, and immune evasion by fusion or delivery into the target cells. Magnified inset depicts EV cargo: viral proteins, genomes, host factors, glycoproteins, and miRNAs. These EVs can also serve as therapeutic carriers or reservoirs for biomarkers, depending on the host and viral status.

**Figure 3:**
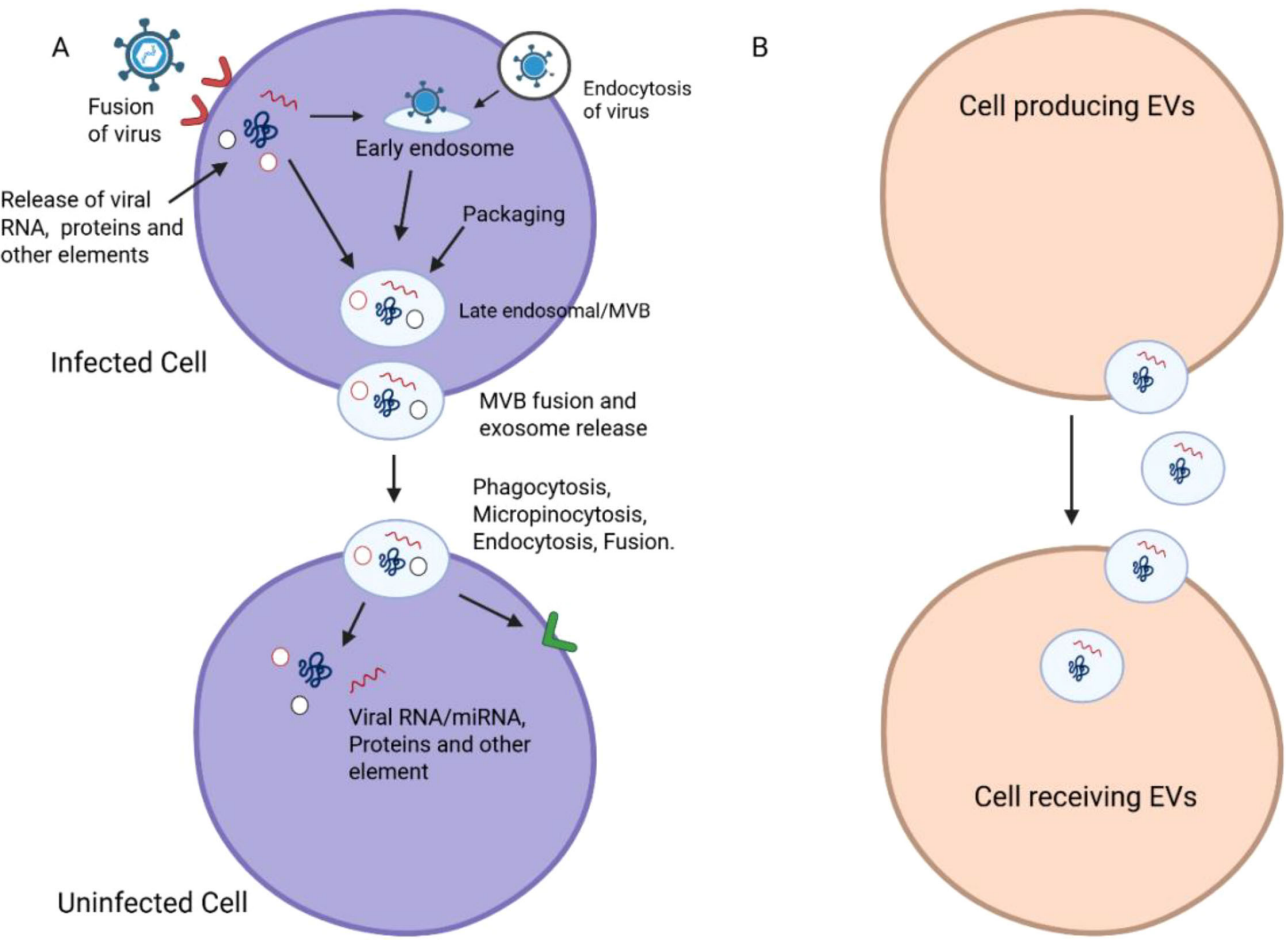
Mechanisms of EV-mediated viral transmission. **(A)** Viral entry in the host cell is depicted schematically as a fusion, endocytosis and phagocytosis/micropinocytosis. The pathways describe the topic of viral RNA/protein and other elements release into the early endosome and its subsequent transport to the late endosomal/early multivesicular bodies (MVBs). **(B)** The following panel depicts viral components encapsulated in EVs, followed by the fusion of MVBs and the release of exosomes. In the process of transmitting viral RNA/miRNA and proteins, we can regard EVs as the vectors that transfer the viruses from infected cells to recipient non-infected cells. Combined, the diagrams emphasize the bipartite function of EVs in viral transmission.

EVs released from virus-infected cells can carry protein molecules encoded by viral genes involved in virus assembly (as shown in **Figures 2**, **3**) and summarized in **Table 2**. When these EVs deliver their virulence-associated contents to recipient cells, they increase the cells’ susceptibility to viral infection. Additionally, the presence of viral protein in EVs can lead to the death of nearby, uninfected immune cells (11), potentially explaining the significant depletion of immune cells observed during the early phases of infection or when the viral load is still low. By transferring viral proteins and cell surface receptors between cells, EVs facilitate the evasion of the host immune response by viruses, thereby suppressing antibody production in lymphocytes and rendering immune cells more susceptible to infection (85).

**Table 2:** Examples of viral genetic material carried by EVs.

Virus	Type of EV	EV cargo	Function in viral pathogenesis	Reference
HIV-1	Exosomes	Viral RNA	Facilitates infection, immune evasion, and viral dissemination	(68)
HCV	Micr Transmission vesicles	Viral RNA, proteins	Enhances replication and cell-to-cell transmission	(69)
EBV	Apoptotic bodies	Viral DNA, miRNAs	Modulates immune response and maintains latency	(10)
Influenza A	Exosomes	Viral RNA, proteins	Enhances viral replication and modulates host immune response	(70)
SARS-CoV-2	Exosomes, Microvesicles	Viral RNA, proteins	Promotes infection, modulates host immune response	(71)
CMV	Exosomes	Viral miRNAs, proteins	Inhibits host immune recognition, promotes viral persistence	(72)
Zika virus	Exosomes	Viral RNA, miRNAs	Facilitates viral dissemination and immune evasion	(9)

EVs, such as exosomes and microvesicles, play a significant role in HIV transmission within the host. Importantly, EVs released from HIV-1-infected cells transfer co-receptors CCR5 and CXCR4 to non-susceptible cells, significantly increasing viral tropism *in vitro* and potentially *in vivo* (68). For instance, EVs from infected peripheral blood mononuclear cells (PBMCs) transfer CCR5 to facilitate macrophage-tropic HIV entry, whereas those from megakaryocytes/platelets transfer CXCR4 to enable X4-tropic viral infection (86). Additionally, exosomes released by dendritic cells can carry HIV particles in CD8-positive compartments, allowing for trans-infection of CD4^+^ T cells (87).

### 5.1 EVs in human immune deficiency virus EVs

HIV-infected cells release EVs that carry the accessory protein Nef, which triggers some of the disease-causing processes: It induces CD4^+^ T cell apoptosis by binding to CXCR4 and inhibiting CD4 and MHC-I surface expression, disrupting adaptive immunity (88). It mediates TNFα release by sequestering ADAM17/TACE, leading to persistent inflammation and reactivation of latent HIV reservoirs (89). Additionally, EVs also carry trans-activation response (TAR) RNA, which, upon delivery to target cells, downregulates pro-apoptotic proteins Bim and Cdk9, promoting cell survival, and increases susceptibility to HIV infection (90). Viral RNA transfer via EVs also activates innate receptors (e.g., TLR8), further promoting chronic inflammation through cytokines like IL-6 and TNFβ (91). EVs containing host-derived pro-inflammatory mediators, including miRNAs miR−155, miR−223, and miR−29a, as well as proteins HIF−1α, have been linked to endothelial activation, immune cell dysfunction, and neuroinflammatory cascades, contributing to HIV-related comorbidities (92).

EVs have the potential to serve as HIV biomarkers and therapeutic agents. Elevated platelet- and endothelial-derived EVs correlate with immune activation and vascular dysfunction in HIV-infected patients. EVs containing Nef protein and TAR RNA have been found in patient plasma even after the administration of combination antiretroviral therapy (cART), making them potential markers for residual viral activity detection (93). Moreover, EVs are also antiviral, some APOBEC3G and cyclic GMP−AMP (cGAMP), which interfere with viral replication and trigger antiviral signaling in target cells (85). This seemingly contradictory role, facilitating HIV transmission on one hand and blocking it on the other, indicates a multifunctional role of EVs that could be harnessed for therapeutic purposes (**Table 3**).

**Table 3:** Role of EVs in enhancing viral replication and pathogenesis across various viral infections.

Virus	EV cargo	Mechanism of action	Pathogenic role	Relevance to host immune response yherapeutic/diagnostic potential	Therapeutic/diagnostic potential	Reference
HIV-1	Nef protein, Tat protein, miRNAs	Modulation of host immune signaling pathways, enhancement of viral transcription	Promotes viral persistence, immune suppression, and evasion	Alter’s innate immune responses reduce T-cell activation	Targeting Nef to reduce viral reservoirs, EVs as biomarkers for viral load in ART-resistant cases	(94)
HCV	Viral proteins (core, NS3, NS5A), host miRNAs	Enhances viral replication by manipulating host machinery, modulates immune responses	Facilitates viral spread across hepatocytes, immune evasion	Inhibits type I IFN responses, promotes chronic infection	EV-mediated delivery of antivirals, potential diagnostic biomarkers for liver damage	(95)
EBV	Viral miRNAs (e.g., BART miRNAs)	Suppresses host antiviral cytokine production, promotes cell proliferation	Maintains viral latency, immune evasion, and contributes to tumorigenesis	Alters immune cell function, promotes immune evasion in B cells	Potential for miRNA-based therapies, EVs as biomarkers for early EBV-related cancer detection	(96)
HBV	Surface proteins (HBsAg), viral DNA	Promotes viral replication and chronic infection, inhibits host immune response	Facilitates viral persistence, liver damage, and immune evasion	Reduces NK cell activation, inhibits cytotoxic T lymphocytes	EVs as potential biomarkers for liver fibrosis and HBV disease progression	(97)
Influenza	Viral proteins (e.g., HA, M1), host RNA	Enhances viral entry into host cells, facilitates immune modulation	Promotes viral spread in respiratory tissues, immune suppression	Inhibits antiviral signaling pathways, interferes with immune cell recruitment	EVs as potential delivery vehicles for antiviral drugs and vaccine	(98)
SARS-CoV-2	Viral proteins (e.g., Spike, N), host miRNAs	EVs aid viral transmission, modulate host immune responses	Enhances viral spread, promotes immune evasion, and cytokine storms	EVs carry viral RNA that alters immune response, promotes lung inflammation	Diagnostic biomarkers for disease severity, potential for EV-based therapeutic interventions	(99)

### 5.2 EVs and hepatitis B virus infection

New studies have confirmed that HBV-infected hepatocytes secrete EVs-CD63^+^/CD81^+^ exosomes containing HBV DNA and intact virions. They demonstrated that these HBV DNA-loaded exosomes infect naive hepatocytes via a ceramide-dependent, ESCRT-independent pathway; these exosomes are resistant to neutralizing antibodies (100). They extended their findings to patient sera and showed that exosomes infect NK cells and inhibit their function (101). Additionally, they demonstrated that exosomal virions carry large surface antigens (LHBs) and enter target cells through NTCP-dependent entry, establishing an alternative entry pathway of HBV. HBV-derived EVs radically reorganize innate immunity. They demonstrated that exosomes from infected hepatocytes increase PD-L1 expression in monocytes and macrophages, inhibiting T-cell activation through PD−1 signaling (101). At the same time, pathogen-induced exosomes both stimulate NK cell activity through MyD88/TICAM-1/MAVS pathways and inhibit IL−12 through microRNAs (e.g., miR−21, miR−29), reducing innate antiviral immunity (71). Proteomics also identified complement factor, lipopolysaccharide-binding protein, and extracellular matrix protein enriched within plasma EVs, linking them to disease progression and potential as biomarkers in chronic hepatitis (**Table 3**) (53).

### 5.3 EVs and hepatitis C virus infection

HCV also hijacks EVs for infectivity and immune evasion. eHAV-like EVs from HCV consist of stable viral RNA-AGO2-HSP90-miR-122 complexes, shielding the RNA from neutralizing antibodies and facilitating target-cell entry (68). EVs also transfer glycoproteins E1/E2 and immunomodulatory miRNAs (e.g., miR−19a, miR−192), triggering fibrosis and inhibiting immune responses through RUNXOR-mediated induction of ARG1, iNOS, STAT3, and ROS-induced T-cell apoptosis. In contrast, EVs secreted by non-parenchymal liver cells (e.g., activated macrophages or LSECs) bearing type I/III interferons or antiviral miRNAs may suppress HCV replication, which suggests opposite roles based on their origin (102). EVs are emerging as promising therapeutic delivery platforms. In HBV, Tenofovir-treated patient serum EVs and macrophage EVs exert antiviral activity, inhibiting HBsAg, HBeAg, HBV DNA, and cccDNA in hepatoma cells—processes mediated by exosomal lncRNA HOTTIP (103). Furthermore, HBsAg-bearing exosomes or adjuvant-modified exosomes elicited robust CTL responses, enabling antigen cross-priming (103). In HCV, EVs carrying let-7f, miR-145, miR-199a, and miR-221delivered from MSCs strongly inhibit viral replication, while strategies targeting EVs release pathway (e.g., ESCRT, ceramide) also reduced HCV infection *in vitro* (100).

### 5.4 EVs and SARS-CoV-2 infection

EVs resemble enveloped viruses in size, composition, and biogenesis which makes conventional separation methods challenging (104). This biophysical mimicry has been exploited for therapy: ACE2-positive EVs (ACE2-EVs), either endogenous or genetically modified, act as decoys by binding the SARS-CoV-2 spike protein and blocking viral attachment to host cells (105). It has been demonstrated that TMPRSS2-enhanced ACE2-extracellular vesicles (ACE2-EVs) neutralize spike-pseudotyped lentiviruses with significant efficacy, which is more potent than the soluble form. Moreover, circulating ACE2-EVs from COVID-19 patients neutralized wild-type and variant strains (α, β, δ) *in vitro*, and protected hACE2-transgenic mice against lung injury (106). Several studies have designed ACE2-expressing EVs as antiviral treatments, showing that intranasal administration of engineered EVs−ACE2 profoundly blocked SARS−CoV−2 pseudovirus infection in mice (107). Nanodecoys, as reported in a study that included ACE2 and cytokine−receptor EVs, not only neutralized the virus but also bound inflammatory cytokines such as IL−6 and GM−CSF, thereby reducing pulmonary injury in pneumonia mouse models (108). In another study it is shown that ACE2−EVs are 500–1000 times more potent than spike−EVs for certain strains, although the potency of EV decoys may vary with modified variants such as Omicron (109). ACE2−EVs exhibit significantly higher blocking activity compared to soluble recombinant ACE2—approximately 135−fold higher potency in spike−binding assays, and provide 60-80-fold higher protection in cell culture and animal models (110). Besides their antiviral decoy function, EVs are also used as vaccine vectors. EVs expressing the SARS-CoV-2 spike protein have been employed in preclinical studies, eliciting robust humoral and cellular immune responses in mice, even without the use of adjuvants. EV-mediated antigen diversion improves antigen presentation and neutralizing antibody responses, offering a safer alternative to viral vectors (**Table 3**) (111).

## 6 EVs role in viral infections and immune modulation

**Figure 4:**
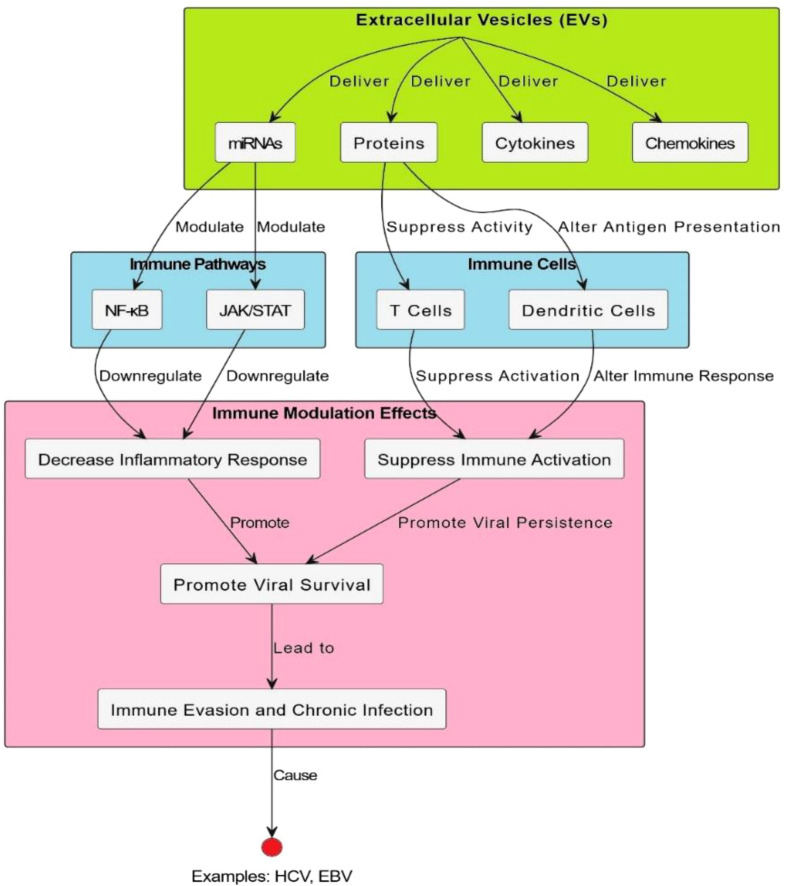
EV-mediated modulation of immune signaling pathways. Extracellular vesicles modulate immune-related signaling pathways. It brings into focus the major interaction events, including the binding of ligand to receptor, cross-linking, and other processes, as well as signal transduction and changes in gene expression. Specifically, EVs are implicated in modulating cellular communication and altering pathways that span from inflammation to antigen presentation and T-cell activation. The schematic also provides an integrative picture of how EVs shape immune responses, including the proposal that they participate in both immune system tolerance and activation. Specific signaling molecules, such as cytokines and receptors, and feedback loops are identified, highlighting the complexity of their involvement in immune regulation and pathology. Moreover, this model emphasizes EVs as therapeutic targets and diagnostic biomarkers in immune-mediated diseases.

EVs show great potential for the prevention and treatment of a wide range of diseases (As shown in **Figure 4**) and detailed in **Table 4**. Despite this promise, their clinical application is hindered by significant challenges, especially regarding low production yields and inconsistent therapeutic effectiveness (112). EVs derived from immune cells play a key role in coordinating and regulating immune responses. Gaining a deeper understanding of their formation, molecular cargo, and functional activities can improve our knowledge of immune system dynamics and lead to new therapeutic possibilities (53). EVs, including MVs and exosomes, have become key facilitators in the spread of viral infections, acting as protective carriers for both enveloped and non-enveloped viruses. Although these vesicles allow viruses to modulate host immune responses, promote their dissemination, and avoid immune detection (113). EVs play an active role in regulating viral infection rather than merely supporting them. EVs can facilitate the non-lytic release of virus from infected cells, allowing the host cell to remain intact while aiding in the infection of new targets (114). Studies have demonstrated that viruses can efficiently infect host cells by utilizing EVs, which enhances their capacity to spread while helping them evade the host’s immune defenses. In addition to these mechanistic insights, quantitative profiling of EV cargo has revealed virus-specific patterns in protein, RNA, and miRNA content. For example, HIV-derived EVs are enriched with Nef protein and TAR miRNA, HBV-associated vesicles carry HBx mRNA and viral DNA, while SARS-CoV-2 EVs contain spike protein and host miRNAs that regulate interferon responses. These quantitative differences highlight how EVs not only carry viral material but also actively shape immune modulation and disease progression (53) (**Table 5**). EVs can also modulate host antiviral signaling. For example, tissue-secreted EVs (ASTEX), such as those containing microRNAs (like miR-16), suppressed mTOR signaling in lung epithelial cells, resulting in decreased viral titers and cytopathic protection. This implies a dual role of EVs: direct antiviral blockade and immune modulation to avoid tissue damage (119).

**Table 4:** Quantitative profiles of EV cargoes in viral infection.

Virus	EV Cargo	Cargo Type	Observed Fold Change/Concentration	Biological Impact	Reference
HIV	Nef protein	Viral protein	↑ ~3.5-fold in EVs from infected cells vs. control	Induces apoptosis in CD4^+^ T cells	(120)
HIV	TAR miRNA	Viral miRNA	~1.6× enrichment in plasma EVs	Activates NF-κB and promotes viral replication	(120)
HBV	HBx mRNA, viral DNA	Viral RNA/DNA	EV DNA >1.5 log copies/mL in serum	Facilitates immune evasion	(121)
HCV	miR-122	Host miRNA	~4-fold increase in serum EVs during infection	Enhances viral replication in liver	(11)
SARS-CoV-2	Spike protein (S1/S2)	Viral protein	Detected at ~100–300 pg/mL in EV fractions	Potential for immune modulation or decoy effect	(107)
SARS-CoV-2	miR-148a, miR-21-5p	Host miRNA	2.5–3.8× higher in COVID-19 patient EVs	Regulates interferon signaling, inflammation	(107)
EBV	BART miRNAs	Viral miRNA	~6-fold higher in EVs from infected cells	Suppresses immune recognition	(122)

The symbol ↑ indicates an increase.

**Table 5:** Role of EVs in immune response during viral infections.

EV mechanism	Immune system component	Impact	Reference
Antigen presentation via MHC molecules	Cytotoxic T Lymphocytes (CTLs), T helper cells	Activation of virus-specific CTLs, initiation of adaptive immune responses through EV-mediated antigen presentation	(115)
EV-mediated cytokine and chemokine release	Macrophages, dendritic cells, NK cells	Activation of innate immune response, enhancement of interferon production, promotion of pro-inflammatory signaling	(116)
EVs carrying viral RNA and proteins	Toll-like Receptors (TLRs), RIG-I, MDA5	Induction of antiviral innate immune pathways (e.g., type I interferon response), activation of antiviral gene transcription	(11)
Immune checkpoint modulation via EVs	Regulatory T cells (Tregs), dendritic cells	Immune suppression through modulation of PD-L1 and other checkpoint molecules on EV surfaces, promoting viral persistence	(117)
EVs carrying host or viral miRNAs	Monocytes, macrophages, epithelial cells	Modulation of host immune response, inhibition of antiviral gene expression, suppression of pro-inflammatory cytokines	(118)
EV-mediated transfer of viral antigens	B cells, dendritic cells, macrophages	Stimulation of antibody production, modulation of B cell responses, contribution to humoral immunity, and long-term viral control	(116)
EV-driven modulation of antiviral immunity	Innate lymphoid cells, NK cells, neutrophils	Enhancement or suppression of antiviral immunity through EV-mediated delivery of modulatory factors like viral proteins or host molecules	(11)

## 7 EVs as biomarkers for viral infections

### 7.1 Diagnostic potential of EVs in detecting viral infections

This makes EVs very promising as diagnostic tools for identifying viral infections. Their ability to encapsulate and transport viral proteins, RNA, and intact viral particles makes EVs an easily accessible source of viral biomarkers. For example, in HBV and HCV-infected patients, extracted EVs carry viral RNA that can be identified using technologies such as PCR (9). Similarly, EVs isolated from HIV and influenza patients contain viral materials that reflect the infection condition. Therefore, they can be very reliable for noninvasive diagnosis (123). Another advantage of EVs is that they can be easily separated from readily accessible biological fluids, such as blood, saliva, and urine, thereby further enhancing their diagnostic utility. Traditional diagnostic technique often relies on biopsy, which is invasive and risky. In contrast, EV-based diagnostics have the potential to be less invasive and more effective in monitoring viral infections. For example, circulating EVs present in the plasma of patients with COVID-19 contain viral RNA and are therefore potentially used as targets for the early detection of SARS-CoV-2 infection (98, 124).

### 7.2 EV-based biomarkers for disease progression and prognosis

The significance of their role as diagnostic agents, EVs, is vital for determining the course and prognosis of viral infections. Generally, the content of the majority of EVs reflects the progression of disease and may be associated with the host immune response or the level of viral spread. For example, in HIV infection, the number of EVs containing viral proteins and host molecules, such as pro-inflammatory cytokines, correlates with the viral load and the degree of immune activation, predicting disease progression (125). In chronic viral infections like HCV, a specific set of miRNAs is trapped inside EVs, including miR-122, which is involved in liver disease progression and fibrosis (68). Its monitoring provides prognostic value, which potentially leads to clinical decisions regarding antiviral therapy or even transplantation at advanced stages. Additionally, research on EVs carrying viral RNA and proteins in emerging viral infections, such as Zika and Ebola, shows that they carry transport viral RNAs and proteins, which have been used to evaluate disease-level patient prognosis (11, 123).

## 8 Therapeutic potential of EVs in viral infections

### 8.1 EVs as vehicles for antiviral drug delivery

EVs, particularly exosomes, offer an exciting delivery system for antiviral drugs because they naturally carry bioactive molecules from one cell to another. EVs enable the direct delivery of therapeutics to infected tissues. They are biocompatible, allowing them to cross biological barriers such as the blood-brain barrier, making them ideal candidates for targeted drug delivery systems (126, 127). EVs can be engineered to transport antiviral agents, including small-molecule drugs, nucleic acids like siRNA and mRNA, and proteins that suppress viral replication or enhance host immune responses (11). For example, exosomes filled with antiviral siRNAs have been studied as a therapeutic strategy to silence key viral genes in HCV infection (128). Loading EVs with therapeutic siRNAs reduces off-target effects and lowers the toxicity of the treatment compared to systemic administration of the drug. Further, new antiviral therapies are being developed with EVs, including the delivery of CRISPR Cas systems aimed at correcting the viral genome in infected cells (**Table 6**) (137, 138).

**Table 6:** EV-based therapies in viral models.

Virus	EV source	EV cargo	Therapeutic application	Outcome	Reference
HIV	MSC-derived EVs	siRNA targeting HIV-1 gag	Antiviral siRNA delivery	↓ HIV RNA expression in PBMC cultures	(129)
	Dendritic cell (DC)-derived EVs	HIV p24 antigen + co-stimulatory molecules	Vaccine immunotherapy	↑ CD8^+^ T cell response and IFN-γ production	(130)
HBV	Hepatocyte-derived EVs	siHBV targeting HBV polymerase	RNA interference therapy	↓ HBV replication and viral antigen *in vitro*	(131)
	Engineered EVs	HBV antigens (HBsAg, HBcAg)	Exosome-based vaccine	↑ Antibody titers, activation of HBV-specific CD4^+^/CD8^+^ T cells in mice	(132)
HCV	MSC-derived exosomes	Anti-HCV siRNA (targeting NS5B)	Gene silencing therapy	↓ HCV RNA levels in infected hepatocytes	(133)
	DC-derived exosomes	HCV NS3/NS4 proteins	Vaccine delivery	↑ T cell response, protection in murine model	(134)
SARS-CoV-2	Engineered EVs expressing ACE2	ACE2 receptors + cytokine scavengers	Antiviral decoy & immunotherapy	↓ Lung injury, ↓ viral titers in ACE2-mice, cytokine neutralization	(135)
	EVs carrying spike protein	Full-length spike or S1 antigen	Vaccine antigen presentation	↑ Neutralizing antibody titers, ↓ weight loss in infected animal models	(107)
Zika Virus	Plasma-derived EVs	Viral RNA, envelope proteins	Diagnostic biomarker development	EV profiling used to distinguish ZIKV-positive from control plasma samples	(136)

The symbol ↓↑ indicates both decrease and increase.

### 8.2 EV-based vaccines and immunotherapies

They have also demonstrated significant potential in developing vaccines and immunotherapies for viral infections. Exosomes derived from either infected or antigen-presenting cells can carry viral antigens, making them valuable tools for stimulating the immune response (139). These EVs trigger both innate and adaptive immune pathways, which are essential for an effective vaccine response. For example, exosomes containing viral proteins can provoke a potent immune response against HIV. Various studies have shown that exosomes pre-loaded with HIV antigens can be stimulate T-cell responses with broad specificity, offering a new approach for vaccine development (69). This method is also being utilized in the development of vaccines against emerging viruses, such as SARS-CoV-2. Such vaccines may include viral spike proteins or other epitopes to trigger the production of neutralizing antibodies (140). In tumor immunotherapy, EVs have already proved their concept by presenting tumor antigens. Similarly, in viral infections, these principles are applied in the use of EV-based vaccines, which now show promise for the inducing long-term immunity (115).

### 8.3 Engineering EVs for targeted therapy

EVs can be engineered for targeted therapies. By modifying the surface proteins of EVs, researchers can design them to target specific virus-infected cells or tissues. This specificity enhances therapeutic outcomes by minimizing the risks of side effects on healthy tissues and targeting antiviral agents where they are needed (139). For example, exosomes can be designed to deliver surface ligands that bind specifically to receptors on infected cells, such as CD4 receptors in HIV-infected cells (139, 141). In this case, targeted delivery increases the effectiveness of the therapeutic agents by delivering their payload directly to the infection site, enhancing the antiviral response.

Additionally, EVs can be engineered to carry immune-modulating molecules, which can boost host immunity against the virus and further expand their therapeutic potential. Another promising area involves developing EVs as platforms for combination therapies. For example, EVs can be loaded with multiple therapeutic agents, such as a combination of antiviral drugs and immune modulators, leading to more effective and adaptable treatments for both chronic and acute viral infections. In case of HBV infections, EVs can deliver siRNAs along with nucleoside analogs to simultaneously inhibit viral replication through a dual blockade of the viral life cycle (**Table 6**) (95).

## 9 Challenges and future perspectives

Although EVs show great potential as biomarkers in viral infections, their clinical use faces several technical and methodological challenges. One major issue is the lack of standardized protocols for the isolation and characterization of EVs. Common techniques, such as ultracentrifugation, size-exclusion chromatography, and immunoaffinity capture, produce EV populations that vary in purity and molecular composition, which hinders reproducibility and comparisons across different laboratories. This inconsistency makes developing universally accepted diagnostic platforms more difficult (142, 143). Another major challenge involves EV heterogeneity. EV populations comprise various subtypes, including exosomes, microvesicles, and apoptotic bodies, which differ in size, origin, and content. Isolating specific subtypes for diagnostic purposes remains difficult because of overlapping physical and biochemical features (143, 144). Additionally, EV samples often contain contaminants such as protein aggregates and lipoproteins, which can hinder the identification of EV-specific biomarkers and functions (145). The sensitivity and scalability of current detection methods are also limited, especially when analyzing low-abundance EV biomarkers in early-stage infections. Although microfluidic platforms, nanotechnology-based tools, and machine learning algorithms offer potential for high-throughput and accurate EV analysis, these approaches still require further improvement for clinical use (144, 146). Understanding EV cargo through integrative omics, proteomics, transcriptomics, and metabolomics can offer deeper insights into their roles in viral pathogenesis and host immune modulation. However, most current findings depend heavily on *in vitro* models with limited validation in *in vivo* systems. Functional assays often lack dose response data or appropriate negative controls, as highlighted in the MISEV2023 guidelines. Additionally, inconsistencies in terminology and classification across studies and databases highlight the need for unified reference standards and broader adoption of the MISEV framework. To bridge these gaps, future research must focus on: (i) improving EV subtype discrimination through advanced imaging and single-vesicle analysis; (ii) developing label-free, scalable isolation technologies; and (iii) enhancing the functional validation of EV roles in infection and immunity. Additionally, EV engineering presents exciting opportunities for targeted drug delivery and the development of antiviral vaccines. Addressing these issues is crucial for the successful translation of EV-based diagnostics and therapeutics into clinical settings.

## 10 Conclusion

EVs are vital mediators of cell-to-cell communication and play diverse roles in the context of viral infections. This review highlighted the multifunctional nature of EVs as modulators of viral pathogenesis, potential diagnostic biomarkers, and therapeutic tools. EVs contribute significantly to the progression of viral disease by enhancing viral infection, facilitating immune evasion, and regulating host immune responses. As such, they serve both as vectors for viral transmission and as promising targets for therapeutic intervention. Key points from this review emphasize the e dynamic role of EVs in shaping the outcomes of viral diseases, due to their ability to encapsulate and transport viral components. EVs not only promote viral replication and dissemination but also reflect the pathological state of infected cells, offering potential utility in diagnostics and disease monitoring. Moreover, advances in EV engineering have opened new avenues for the delivery of antiviral drugs, vaccine development, and the design of targeted therapies. These developments highlight the need to view EVs not just as passive bystanders but as active players in viral life cycle and as tool for medical innovation.
